# Flexor Tendon Tenosynovitis in a Child Following Missed Thorn Prick Injury: A Case Report

**DOI:** 10.1055/s-0045-1802314

**Published:** 2025-02-05

**Authors:** Bipin A. Ghanghurde, Rohan Habbu, Bhupendra S. Avasthi

**Affiliations:** 1 Department of Plastic Surgery, Wadia Children's Hospital, Mumbai, Maharashtra, India; 2 Department of Orthopaedics, Surya Hospital, Santacruz, Mumbai, Maharashtra, India; 3 Department of Pediatrics, Surya Hospital, Santacruz, Mumbai, Maharashtra, India

**Keywords:** thorn prick injury, child, tenosynovitis

## Abstract

Plant thorn synovitis is a frequently missed and neglected injury. We report a delayed case of suspected thorn injury to the left middle finger in a 5-year-old child, which was treated with thorn removal and complete flexor synovectomy to achieve good results. Proper history, clinical examination, and correlated radiological findings can help in the diagnosis. Patients may seek late treatment due to persistent swelling and pain. Antibiotic may be given; however, in a delayed case, its role is debatable. Thorn removal and complete tenosynovectomy offers complete recovery.

## Introduction

Plant thorn synovitis is a frequently missed and neglected injury in the developing world. 1 2 A complete history is vital in establishing the diagnosis. We report a 2-month delayed case of suspected thorn injury to the left middle finger in a 5-year-old child with pain and inability to make fist. The patient was treated with thorn removal and complete flexor tenosynovectomy to achieve good results. Radiographic imaging with magnetic resonance imaging (MRI) can be helpful for diagnosis. 3 The parents' consent was obtained before reporting this case and ethical approval has been taken.

## Case Report

**Fig. 1:**
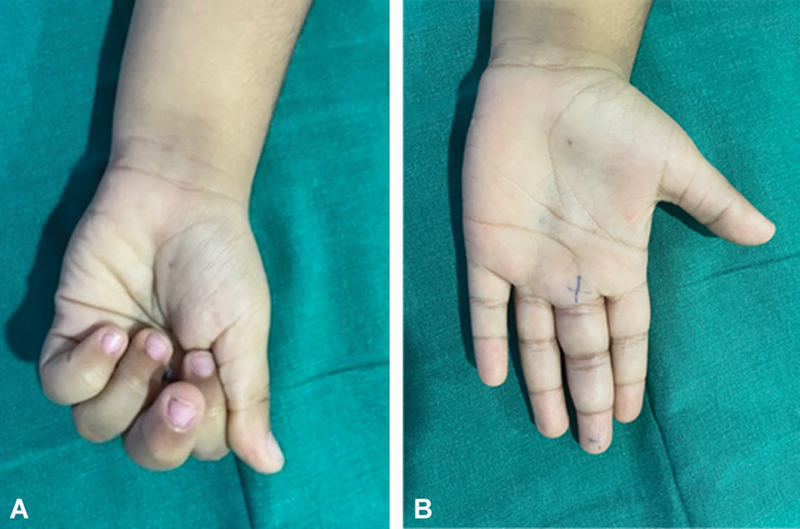
( **A** ) Preoperative swelling of the left middle finger. ( **B** ) Preoperative inability to flex the left middle finger.

**Fig. 2:**
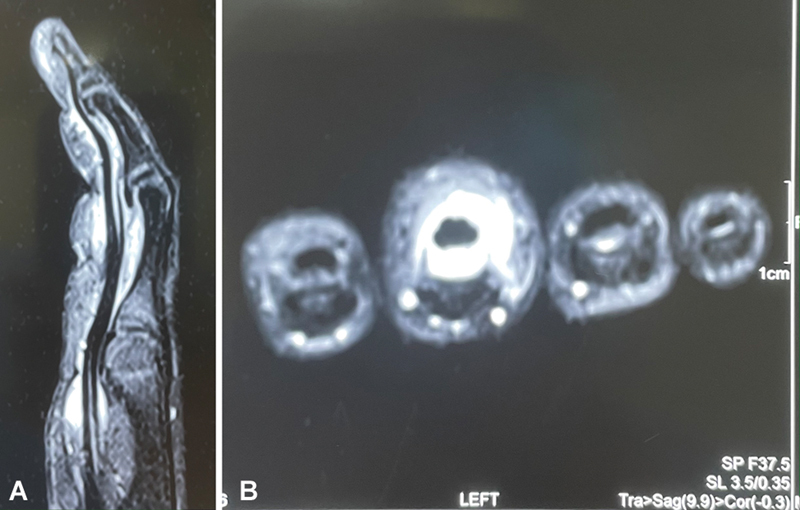
( **A** ) Magnetic resonance imaging (MRI) T2-weighted sagittal section showing flexor tenosynovitis. ( **B** ) MRI T2-weighted coronal section showing the synovium engulfing the tendon.

**Fig. 3:**
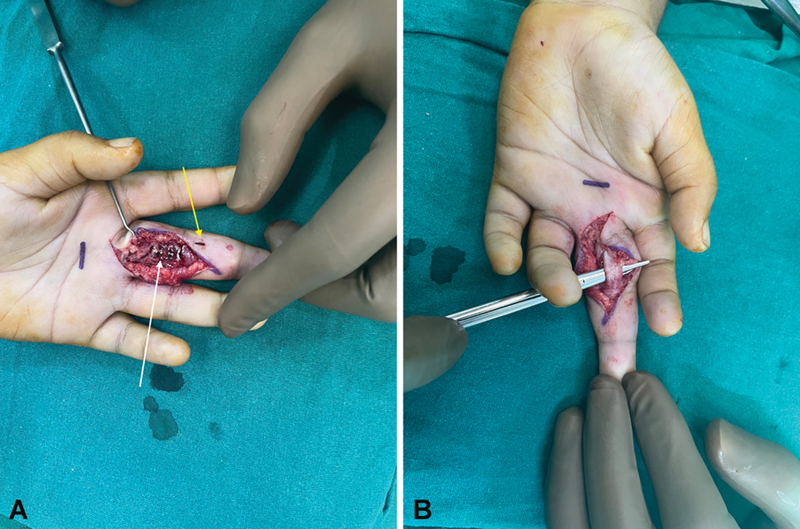
( **A** ) Intraoperative image showing dirty tissue engulfing the flexor tendon ( *white arrow* ) and thorn ( *yellow arrow* ). ( **B** ) Intraoperative image after complete tenosynovectomy.

A 5-year-old right-hand-dominant child presented with pain, swelling, and inability to make fist due to restricted flexion of the left-hand middle finger ( Fig. 1 ). The child's mother gave history of a suspected thorn prick injury 2 months before presentation followed by pain, swelling, and inability to move the finger. The pain reduced over a week following the use of empirical broad-spectrum antibiotics and analgesics; however, discomfort and swelling persisted over the injured finger. At 2 months after injury, the child was referred to us with pain, mild swelling, and restricted flexion of the left middle finger; the range of motion at the proximal interphalangeal joint was10 to 40 degrees, and there was tenderness along the volar side of the proximal interphalangeal joint of the finger. The child was unable to grip while playing a monkey bar. Radiographs were normal. MRI showed that there was flexor tenosynovitis ( Fig. 2 ). The blood counts, white blood cell (WBC), C-reactive protein (CRP), and erythrocyte sedimentation rate (ESR), were within normal limits. After obtaining proper consent of the parents, the finger was explored with modified Brunner's incision and it was found that the flexor tendons in zones 2 and 3 were completely engulfed with a dark reddish synovium, which was completely excised and, in the process, the foreign body was extracted ( Fig. 3 ). The tissue was sent for histopathology, tuberculosis culture, Gram stain, tissue culture, and fungal culture. Postsurgery, a dorsal slab and elevation were given for 10 days. Following suture removal, active assisted range of movements were begun.

## Results

**Fig. 4:**
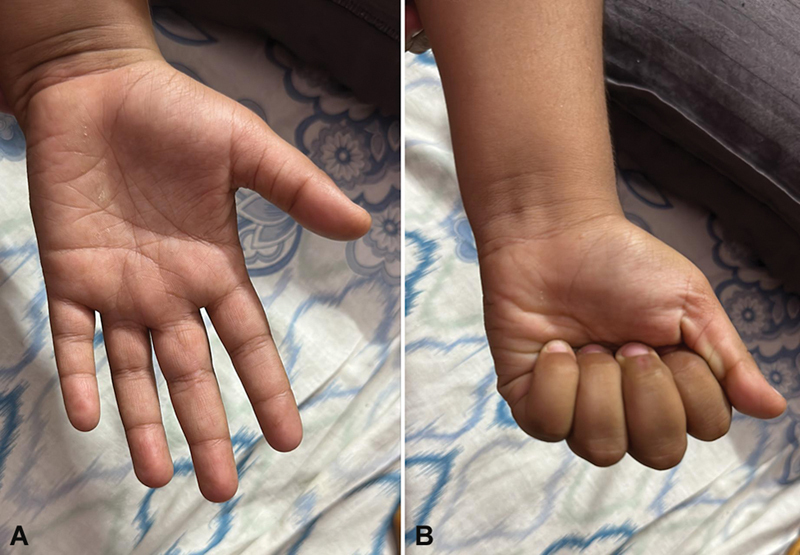
( **A** ) At 2 years of follow-up showing no swelling and full extension of the finger. ( **B** ) At 2 years of follow-up showing complete flexion and making a fist.

Histopathology reported as hyperplastic vascularized synovium with chronic inflammation and the culture bacteria, tuberculosis, and fungus were negative. Two months after the surgery, the patient had pain relief, no swelling and full flexion, and extension of the finger could be achieved. Two years after surgery and at the time of the last follow-up, the child performed all daily activities and was able to play with the monkey bar without pain or discomfort ( Fig. 4 ).

## Discussion

Plant thorn synovitis is one of the rare causes of flexor tenosynovitis, 3 with few authors reporting it. 2 3 4 5 6 7 We report plant thorn flexor synovitis in a 5-year-old child, which according to our literature search is very uncommon in a child.

Pyogenic flexor tenosynovitis is a fulminant infection in individuals with comorbidities, especially diabetes mellitus, hematogenous spread, or local inoculation due to a bite or a stab. 8 In this patient, there was history of thorn prick, but typical signs of Kanavel were negative, which can be attributed to the antibiotic course; however, fusiform finger swelling can be correlated to the chronicity of inflammation. 9

There are reports of Enterobacteriaceae group organisms, such as *Pantoea agglomerans* , being found in penetrating injuries caused by plants or thorns. 10 However, due to the indolent nature of this pathogen and low clinical suspicion, it is missed. We were unable to isolate any organism after 2 months possibly due to delayed culture and use of empirical antibiotic. Some authors have recommended the use of empirical antibiotics in acute cases. 11 12 Our patient was given antibiotics, as this was a routine practice in the emergency department (telephonic conversation with the pediatrician) and it did not target any organism.

Plant thorn synovitis can also be aseptic. 2 3 4 Aseptic tenosynovitis following a thorn injury is often forgotten. The symptoms are mild but nagging, which is why there is a delay in presentation. 2 Irritation due to the thorn or foreign body can be considered a cause of pain. The case of a child may be considered aseptic as no organisms were cultured and blood reports were within normal limits.

The treatment options for a plant thorn tenosynovitis include only removal of foreign body, partial tenosynovectomy, and complete synovectomy. 2 Complete synovectomy as a treatment of choice has also been supported by De Smet and Fabry, 1 Baskar et al, 2 and Doig and Cole 13 over partial tenosynovectomy as there is no recurrence. We performed exploration and complete tenosynovectomy of the flexor digitorum superficialis and the flexor digitorum profundus ( Fig. 3B ). The child recovered well within 2 months and at 2 years after surgery and at the time of the last follow-up, she was able to grip well and perform all activities at play.

Immediately after injury, a proper history is vital and after radiological confirmation, exploration should be advised to decompress the compartment and remove any foreign bodies. Empirical broad-spectrum antibiotics for 2 weeks are recommended to take care of indolent infections.

## Conclusion

Flexor tenosynovitis following thorn injuries in children is rare and diagnosis may be missed. However, proper history, clinical examination, and correlated radiological findings can help in the diagnosis. Patients may seek late treatment due to persistent mild swelling, pain, and discomfort. Thorn removal and complete tenosynovectomy offers complete recovery.
